# Valproic Acid Synergizes With Cisplatin and Cetuximab *in vitro* and *in vivo* in Head and Neck Cancer by Targeting the Mechanisms of Resistance

**DOI:** 10.3389/fcell.2020.00732

**Published:** 2020-08-17

**Authors:** Federica Iannelli, Andrea Ilaria Zotti, Maria Serena Roca, Laura Grumetti, Rita Lombardi, Tania Moccia, Carlo Vitagliano, Maria Rita Milone, Chiara Ciardiello, Francesca Bruzzese, Alessandra Leone, Ernesta Cavalcanti, Rossella De Cecio, Giuseppina Iachetta, Salvatore Valiante, Franco Ionna, Francesco Caponigro, Elena Di Gennaro, Alfredo Budillon

**Affiliations:** ^1^Experimental Pharmacology Unit-Laboratory of Naples and Mercogliano (AV), Istituto Nazionale Tumori IRCCS “Fondazione G. Pascale”, Naples, Italy; ^2^Laboratory Medicine Unit, Istituto Nazionale Tumori IRCCS “Fondazione G. Pascale”, Naples, Italy; ^3^Pathology Unit, Istituto Nazionale Tumori IRCCS “Fondazione G. Pascale”, Naples, Italy; ^4^Department of Biology, University of Naples Federico II, Naples, Italy; ^5^Maxillo-facial & ENT Surgery Unit, Istituto Nazionale Tumori IRCCS “Fondazione G. Pascale”, Naples, Italy; ^6^Head and Neck Medical Oncology Unit, Istituto Nazionale Tumori IRCCS “Fondazione G. Pascale”, Naples, Italy

**Keywords:** HDAC inhibitor, valproic acid, cisplatin, cetuximab, epidermal growth factor receptor, head and neck squamous cell carcinoma

## Abstract

Recurrent/metastatic head and neck squamous cell carcinoma (R/M HNSCC) is a devastating malignancy with a poor prognosis. The combination of cisplatin (CDDP) plus cetuximab (CX) is one of the standard first-line treatments in this disease. However, this therapeutic regimen is often associated with high toxicity and resistance, suggesting that new combinatorial strategies are needed to improve its therapeutic index. In our study, we evaluated the antitumor effects of valproic acid (VPA), a well-known antiepileptic agent with histone deacetylase inhibitory activity, in combination with CDDP/CX doublet in head and neck squamous cell carcinoma (HNSCC) models. We demonstrated, in HNSCC cell lines, but not in normal human fibroblasts, that simultaneous exposure to equitoxic doses of VPA plus CDDP/CX resulted in a clear synergistic antiproliferative and pro-apoptotic effects. The synergistic antitumor effect was confirmed in four different 3D-self-assembled spheroid models, suggesting the ability of the combined approach to affect also the cancer stem cells compartment. Mechanistically, VPA enhanced DNA damage in combination treatment by reducing the mRNA expression of ERCC Excision Repair 1, a critical player in DNA repair, and by increasing CDDP intracellular concentration *via* upregulation at transcriptional level of CDDP influx channel copper transporter 1 and downregulation of the ATPAse ATP7B involved in CDDP-export. Valproic acid also induced a dose-dependent downregulation of epidermal growth factor receptor (EGFR) expression and of MAPK and AKT downstream signaling pathways and prevent CDDP- and/or CX-induced EGFR nuclear translocation, a well-known mechanism of resistance to chemotherapy. Indeed, VPA impaired the transcription of genes induced by non-canonical activity of nuclear EGFR, such as cyclin D1 and thymidylate synthase. Finally, we confirmed the synergistic antitumor effect also *in vivo* in both heterotopic and orthotopic models, demonstrating that the combined treatment completely blocked HNSCC xenograft tumors growth in nude mice. Overall, the introduction of a safe and generic drug such as VPA into the conventional treatment for R/M HNSCC represents an innovative and feasible antitumor strategy that warrants further clinical evaluation. A phase II clinical trial exploring the combination of VPA and CDDP/CX in R/M HNSCC patients is currently ongoing in our institute.

## Introduction

Head and neck squamous cell carcinoma (HNSCC) is the seventh most common cancer and a leading cause of cancer-related mortality worldwide^1^. Despite the development of multiple integrated approaches such as surgery, chemotherapy, radiotherapy, the antiepidermal growth factor receptor (EGFR) monoclonal antibody cetuximab (CX), and, recently, the use of immune checkpoint inhibitors (ICI), have improved outcome, the long-term survival rate is still poor, particularly for recurrent or metastatic disease that develops in more than 65% of patients (Chow, 2020). Cisplatin (CDDP) represents a standard of care in locally advanced HNSCC in combination with radiotherapy and in recurrent and metastatic (R/M) disease in combined systemic regimens (Chow, 2020).

Epidermal growth factor receptor overexpression has been observed in about 90% of HNSCC specimens, with the exception of human papillomavirus (HPV)-positive tumors, and correlates with poor disease-free and overall survival, metastasis, and resistance to CDDP-based chemotherapy and radiotherapy (Burtness et al., 2013). The approval of CX introduced the first targeted therapy in HNSCC, thereby defining a new standard of care in first-line treatment of recurrent/metastatic head and neck squamous cell carcinoma (R/M HNSCC) accordingly to the EXTREME phase-III trial that demonstrated significantly improved survival and response rate by adding CX to platinum-based chemotherapy, compared to chemotherapy alone (Vermorken et al., 2008)^2^. Interestingly, no other EGFR-blocking agent, including the anti-EGFR monoclonal antibody panitumumab, has matched the result of the EXTREME trial (Szturz and Vermorken, 2017).

Recently, the anti-programed cell death protein 1 (PD-1) ICI pembrolizumab and nivolumab, in monotherapy, improved overall survival compared with standard of care, in patients with R/M HNSCC that progressed during or after platinum-based chemotherapy (Ferris et al., 2016; Cohen et al., 2019). Moreover, the FDA also approved pembrolizumab for the first-line treatment of R/M HNSCC patients in combination with platinum and 5-fluorouracil (5FU) and in monotherapy in programmed cell death ligand 1 (PD-L1) expressing patients (Burtness et al., 2019)^3^. Nevertheless, overall more than 80% of R/M HNSCC patients do not respond or progress after response to ICI treatment (Burtness et al., 2019; Chow, 2020).

Therefore, identifying new therapeutic approaches and/or optimize current treatments to improve efficiency and survival of HNSCC patients is an urgent clinical need.

Epigenetic dysregulations, including histone modifications, are hallmarks of cancer and have been reported in HNSCC (Castilho et al., 2017). Histone deacetylase inhibitors (HDACi) are a family of antitumor agents that by targeting histone and non-histone proteins deacetylation, can modulate gene expression and cellular functions, regulating different altered pathways in cancer, such as apoptosis, cell cycle, and DNA repair (Budillon et al., 2007). Our group and many others have demonstrated the synergistic antitumor activity of HDACi in combination with a large number of structurally different anticancer agents (Suraweera et al., 2018; Roca et al., 2019), including anti-EGFR agents (Bruzzese et al., 2009, 2011; Leone et al., 2015; Citro et al., 2019; He et al., 2019) and CDDP (Piro et al., 2019).

Valproic acid (VPA), a safe and low cost generic antiepileptic and mood stabilizer agent, has HDAC inhibitory activity and anticancer properties, with good safety profile compared with other HDACi, thereby representing a good candidate to be tested in combination therapy in cancer patients (Chateauvieux et al., 2010; Avallone et al., 2014). Valproic acid has been evaluated in combination with platinum-based drugs in many cancer cell models, including HNSCC (Diyabalanage et al., 2013). Valproic acid has been also tested in cancer patients and several antitumor clinical trials are ongoing^4^. A good tolerability and encouraging tumor responses of VPA in combination treatment were observed in ongoing clinical trials launched in our institute (Caponigro et al., 2016; Budillon et al., 2018)^5^.

In the present study, we examined, for the first time, the antitumor efficacy of a three-drug regimen combining VPA, with the CDDP/CX doublet in HNSCC models. Indeed, based on recent clinical trials CDDP/CX combination offers a valid option compared to the EXTREME regimen (Bossi et al., 2017), thus representing a potential backbones for combinations with new and emerging agents. We demonstrated the ability of VPA to induce synergistic antitumor effect, in combination with CDDP/CX, in both *in vitro* and *in vivo* models by increasing DNA damage and impairing the main mechanisms of resistance against both CDDP and CX. On these bases, a phase II clinical study of VPA in combination with CDDP and CX in R/M HNSCC patients, is ongoing in our institute (Caponigro et al., 2016).

## Materials and Methods

### Cell Lines

Head and Neck squamous cancer carcinoma cell lines FaDu, SCC9, and Normal fibroblasts BJ-hTERT, were purchased from the American Type Culture Collection (ATCC, Rockville, MD, United States); Cal27 cell lines were kindly provided by Dr. J.L. Fishel (Centre A Lacassagne, Nice, France). The green fluorescent protein^+^/luciferase^+^ (GFP^+^/Luc^+^) Cal27 cell line were obtained by lentiviral infection as described previously (Piro et al., 2019). HOC313 and ZA cell lines were kindly provided by Dr. N. Tsuchida (Faculty of Medicine, Saitama Medical University, Saitama, Japan) (Tadokoro et al., 1989).

Cal27, ZA, SCC9, HOC313, and BJ-hTERT cells were cultured in Dulbecco’s modified Eagle’s medium (DMEM), whereas FaDu were cultured in RPMI-1640 medium. All media were supplemented with 10% heat-inactivated fetal bovine serum (for ZA cells media was supplemented with 20% of heat-inactivated fetal bovine serum), 50 units/mL penicillin, 500 μg/mL streptomycin, and 4 mmol/L glutamine. Cultures were maintained in a humidified atmosphere of 95% air and 5% CO_2_ at 37°C.

All cell lines were regularly inspected for mycoplasma. The cells have been authenticated with short tandem repeat profile generated by LGC Standards.

### Reagents

All media, sera, antibiotics, and glutamine for cell culture were from Lonza (Basel, Switzerland). Primary and secondary antibodies are listed in Supplementary Material.

### Drugs

Valproic acid (2-propylpentanoic acid, VPA) was purchased from Enzo Life Sciences and dissolved in sterile water; Cisplatin (*cis*-Diamineplatinum(II) dichloride, CDDP) is from Sigma-Aldrich and dissolved in sterile phosphate buffered saline (PBS); Cetuximab (Erbitux^®^, CX) was bought from Merck Serono as solution. Stock solutions were diluted to appropriate concentrations in culture medium before addition to the cells.

### Cell Proliferation Assay and Drugs Combination Studies

Cell proliferation was measured in 96-well plates in cells untreated and treated with VPA, CDDP, or CX as single agents, or in combination, using a spectrophotometric dye incorporation assay (Sulforhodamine B) (Bruzzese et al., 2013).

Drugs combination studies were based on concentration-effect curves generated as a plot of the fraction of unaffected (surviving) cells versus drug concentration after 96 or 144 h treatment. In detail, for VPA/CDDP combination studies, the cells were treated with equipotent doses of the two agents (50:50 cytotoxic ratio) for 96 h in two different sequences of treatment: simultaneously or sequentially (24 h delay between the two agents). For VPA/CDDP/CX combination studies, 50:50 cytotoxic ratio of VPA and CDDP plus fixed dose of CX for 144 h were tested in three different sequences of treatment, with CDDP/CX considered as single drug.

Synergism, additivity and antagonism were quantified after the evaluation of the combination index (CI), which was calculated by the Chou-Talalay equation with CalcuSyn software (Biosoft, Cambridge, United Kingdom), as described elsewhere (Terranova-Barberio et al., 2017). A CI < 0.8, CI < 0.9, CI = 0.9–1.1, and CI > 1.1 indicated a strong synergistic, synergistic, additive, and antagonistic effect, respectively. The DRI (dose reduction index) determines the magnitude of dose reduction allowed for each drug when given in combination, compared with the concentration of a single agent that is needed to achieve the same effect.

### Spheroid-Forming Assay

Spheroids were cultured in Sphere Medium (DMEM/F12 supplemented with BSA, glucose, heparin, FGF, EGF, neuronal cell culture B27, insulin). The cells (40,000 cells/mL) were plated in low-attachment multi-well plates and treated with indicated drugs. Times and doses of treatments are described in results section. Spheroids were scored with CellTiter-Glo^®^ 3D Cell Viability Assay (Promega, Madison, WI, United States).

### Light Sheet Fluorescence Microscopy (LSFM)

Spheroids were embedded in 1% low-melting agarose solution (wt/vol) in glass capillaries and mounted in the Z1 Light Sheet Fluorescence Microscope (Zeiss). The microscope imaging chamber was filled with distilled water. Samples were overviewed by led light illumination, then illuminated by 488-nm laser source and fluorescence emission at 525 nm was detected through a 20X NA = 1 Zeiss water immersion objective; images were acquired by a PCO. Edges CMOS water cooled camera. Data were obtained by ZEN 2012 software (Zeiss) analysis and Fiji software v.1.48.

### Western Blot Analysis

Immunoblotting with the indicated antibodies was performed as described elsewhere (Bruzzese et al., 2006). Densitometric analysis was performed using NIH ImageJ software.

### RNA Isolation, RT-PCR Assays, and Real-Time PCR

RNA was isolated by TRizol (Invitrogen) reagent as previously described (Carbone et al., 2017). Real-Time PCR by ABI Prism 7900 HT Sequence Detection System (Applied Biosystems) was performed using specific TaqMan probes. The ERCC Excision Repair 1 (ERCC1), Thymidylate Synthetase (TYMS), Cyclin D1 (CCND1), Copper transporter -Solute Carrier Family 31-Member 1 (CTR1or SLC31A1) and ATPase Copper Transporting Beta (ATP7B) relative mRNA expression levels were calculated using the 2^–ΔΔCt^ method and were normalized to that of the endogenous control.

### Caspase 3/7 Bioluminescence Assay

The cells (5,000 cells/well) were seeded into a 96-well plate and treated for 24 h as indicated. The combined caspase 3/7 activity was analyzed in triplicates using the Caspase-Glo^®^ 3/7 assay (Promega, Madison, WI, United States) according to the manufacturer’s protocol with some modifications. Briefly, after aspirating the medium, Caspase-Glo reagent and the samples were incubated at room temperature for 30 min and the caspase activity was assessed by measuring the luminescence in a multilabel reader (VICTOR X4 2030 PerkinElmer, Waltham, MA, United States).

### Immunofluorescence Detection of γH2AX Foci

DNA damage was measured by immunofluorescence assay using a primary antibody specific for γH2AX and a secondary Alexa Fluor^®^ 594 antibody, as previously described (Terranova-Barberio et al., 2017). The nuclei were stained using DAPI (40,6-diamidin-2-phenylindole). The images were obtained using a confocal microscope Zeiss LMS510 (Zeiss).

### Heterotopic and Orthotopic *in vivo* Experiments

Female, five-week-old, CD1 athymic mice (Charles River, Wilmington, MA, United States) were acclimatized in the Animal Care Facility of CROM (Centro Ricerche Oncologiche Mercogliano) “Fondazione G. Pascale” – IRCCS. Both heterotopic and orthotopic *in vivo* experiments were performed in compliance with institutional guidelines and regulations (Directive 2010/63/EU; Italian Legislative Decree DLGS 26/2014) and after approval from the appropriate institutional review board and the Italian Ministry of Health (N. 865/2015-PR). After one week of acclimatization, cells were injected.

#### Heterotopic Model

Cal27 cells (6 × 10^6^) diluted in 200 μL [1/1 PBS/Matrigel GF] (Becton Dickinson)] were injected subcutaneously (s.c) in the flank regions of the mice. In two mice/group the cells were injected in both flanks in order to perform a pharmacodynamics analysis. When the tumors became palpable, the mice were randomized into four experimental groups (*n* = 7). The mice were treated intraperitoneally (i.p.) with VPA (200 mg/kg melted in water and diluted in a physiological solution, five times a week for two weeks), and/or CDDP (1 mg/kg melted in PBS 1X and diluted in a physiologic solution) based on previous reports (Terranova-Barberio et al., 2016; Piro et al., 2019) and CX (1 mg/kg diluted in a physiologic solution) based on a pilot study (data not shown) and previous reports (Jedlinski et al., 2017). The mice in the control group were treated with drugs vehicles. CDDP and CX were injected three times a week for 2 weeks. Tumor volume (mm^3^), tumor growth delay (TGD) and the percent change in tumor volume from the time of initial treatment to the end of the study were evaluated as described before (Terranova-Barberio et al., 2016).

#### Orthotopic Model

GFP^+^/luc^+^-transfected Cal27 cells (6 × 10^4^) suspended in 50 μL of PBS were injected directly into the anterior tongue. Four days after injection, mice (*n* = 10) were randomized into four experimental groups. The mice were treated intraperitoneally (i.p.) with VPA (200 mg/kg melted in water and diluted in a physiological solution, five times a week for 3 weeks), and/or CDDP (1 mg/kg melted in PBS 1X and diluted in a physiologic solution) based on previous reports (Terranova-Barberio et al., 2016; Piro et al., 2019) and CX (1 mg/kg diluted in a physiologic solution) based on a pilot study (data not shown) and previous reports (Jedlinski et al., 2017). CDDP and CX were injected three times a week for 3 weeks. The mice in the control group were treated with drugs vehicles. The tumor volumes were monitored by IVIS Imaging (PerkinElmer) and the signal intensity (photons/second) was quantified using the Living Image Software 4.1 (PerkinElmer).

#### Biochemistry Tests

At the end of treatment (day 21) three mice of each group were scarified and whole blood samples were collected by intracardiac puncture. The blood was centrifuged at 2,500 rpm for 10 min to separate the serum. Biochemistry evaluation of glutamate oxaloacetate transaminase (GOT) activity, glutamate pyruvate transaminase (GPT) activity, and creatinine levels were performed by a COBAS analyzer (Roche).

### Immunohistochemistry on Xenograft Tumor Samples

We evaluated the expression of EGFR, histone-H3 acetylation (AcH3) and Ki67 by immunohistochemistry (IHC) on formalin fixed paraffin embedded (FFPE) tumor samples derived from mice sacrificed at the indicated time points. Briefly, the sections were incubated with primary antibodies and then with biotin-conjugated secondary antibodies before incubation with specific streptavidin HRP-conjugated tertiary antibody (Dako). Peroxidase reactivity was visualized using a 3,3’-diaminobenzidine (Abcam). A single pathologist (R D.C.) performed a blinded analysis of the slides.

### Statistical Analysis

The results of the *in vitro* cell viability assays are expressed as the mean for at least three independent experiments, which were conducted in quadruplicate (±SD) and the statistical significance was determined by Tukey’s multiple comparisons test. The results of the *in vitro* 3D cell viability assays were expressed as the mean for at least three independent experiments, which were conducted in quadruplicate (±SEM) and statistical significance of differences among the groups was determinate by one-way ANOVA, followed by unpaired t-test with a threshold set as *p* < 0,05. The results of the apoptotic analysis and qRealTime PCR experiments were expressed as the mean for at least three independent experiments (±SEM), and the statistical significance was determined by Tukey’s multiple comparisons test.

Representative results from a single experiment of western blot and immunohistochemistry were presented; additional experiments yielded similar results.

Statistical significance in the differences of tumor growth *in vivo* was determined by Tukey’s multiple comparisons test or Welch’s *t*-test.

Comparison among groups in the survival data was made using the log-rank test.

All statistical evaluations were performed with GraphPad Prism 7 software. A *p* < 0.05 was considered to be statistically significant (^∗^*p* ≤ 0.05, ^∗∗^*p* ≤ 0.005, ^∗∗∗^*p* ≤ 0.001, ^****^p ≤ 0.0001).

## Results

### VPA Potentiates CDDP/CX Antitumor Effect by Targeting the Cancer Stem Cells Compartment and Increasing DNA Damage

First, we evaluated the antiproliferative effect of either VPA, CDDP, or CX, as single agents in HNSCC cell lines, Cal27, FaDu, ZA, HOC313 and SCC9 (Supplementary Table S1), characterized by different molecular features (Supplementary Table S2). FaDu cells are the most sensitive to all drugs tested and particularly to CX, with the half maximal inhibitor concentration value measured after 96h of treatment (IC_50_^96h^) of 4.8 μg/mL (Supplementary Table S1). Conversely, all the other cell lines are clearly resistant to CX and the IC_50_^96h^ value was not achieved (Supplementary Table S1).

**FIGURE 1:**
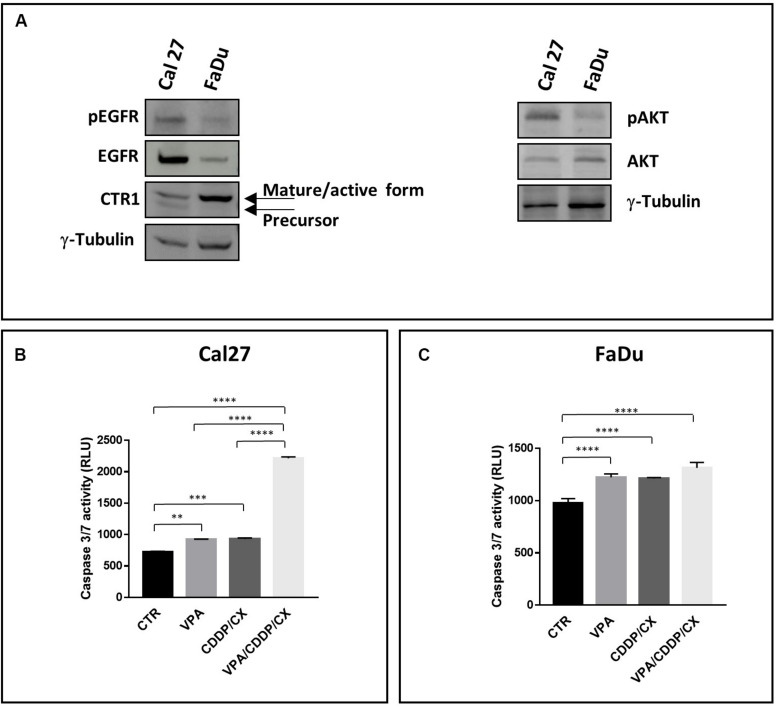
Distinct features of Cal27 and FaDu cells and pro-apoptotic effects induced by VPA/CDDP/CX in HNSCC cells. **(A)** Phospho-EGFR (pEGFR), EGFR, CTR1, phospho-AKT (pAKT), AKT, protein basal levels were determined by western blot in Cal27 and FaDu HNSCC cell lines; γ-tubulin was used as protein loading control. Caspase 3/7 activity was evaluated in Cal27 **(B)** and FaDu **(C)** cells untreated or treated with VPA and/or CDDP/CX at IC_50_^96h^ doses for 24 h, by luminescence assay Results are shown as mean ± SD. Statistical analysis: Tukey’s multiple comparisons test, ^∗∗^*p* ≤ 0.005, ^∗∗∗^*p* ≤ 0.001, ^****^p ≤ 0.0001.

In FaDu cells, both RAS and PI3K are not mutated (Supplementary Table S2), and consistently they express low basal levels of activated AKT (pAKT) (Figure 1A). Moreover, FaDu cells also express lower basal level of EGFR protein and activated EGFR (pEGFR) compared to Cal27 cells, as shown by both western blotting and flow cytometry analysis (Figure 1A and Supplementary Figure S1). Altogether, these data could explain the high sensitivity of FaDu cells to CX treatment. Notably, Cal27, described as CDDP-resistant cells (Piro et al., 2019), expressed significant lower levels of the CDDP influx channel CTR1 active form compared to FaDu, in agreement with a twofold increased CDDP IC_50_^96h^ value (Figure 1A).

We next investigated the antitumor effect of VPA in combination with CDDP at equipotent doses (50:50 ratio) by exploring simultaneous or sequential schedule (24h delay between the two agents) for 96h and calculating CIs at 50% (CI_50_), 75% (CI_75_), and 90% (CI_90_) of cell lethality (Supplementary Table S3). In both Cal27 and FaDu cells, we observed additive/synergistic effect with CI_50_ values ≥ 0.9 and CI_75_ and CI_90_ consistently <0.9, independently of the schedule used, confirming the antitumor synergistic interaction between HDACi and CDDP reported by us and other groups (Rikiishi et al., 2007; Piro et al., 2019; Wawruszak et al., 2019).

We then explored VPA in combination with the CDDP/CX doublet. Evaluating the antiproliferative effect of a monoclonal antibody in short-term cell culture is a particularly challenging task; therefore, cells were treated for 144 h with equipotent doses of VPA and CDDP as described above, either simultaneously or sequentially, and a fixed dose of CX, depending on cell line (Table 1). We obtained consistent synergistic anti-proliferative effects of the triple combination, in all cell lines with CI_50_ and CI_75_ below 0.8 in the majority of the cases, independently of the schedule used. Significantly, in hTERT-immortalized foreskin fibroblast cell line BJ-hTERT we observed antagonist effects (Table 1), suggesting a selective synergistic effect of the triple combination on tumor cells.

**TABLE 1:** Antiproliferative effect induced by VPA/CDDP/CX combination according to the different schedules of exposure in Cal27, FaDu, ZA, HOC313, SCC9, and BJ-hTERT cell lines.

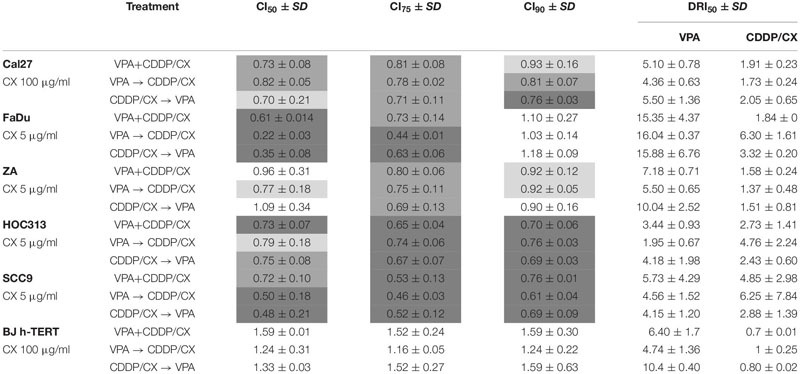

*Synergistic interaction between VPA and CDDP/CX evaluated in Cal27, FaDu, ZA, HOC313, SCC9 and BJ-hTERT cells by calculation of CI. CI values calculated at 50, 75, and 90% of the cell lethality (CI_50_, CI_75_, and CI_90_, respectively) by CalcuSyn software after 144 h of treatment (Mean ± SD from at least three separate experiments performed in quadruplicate). CIs smaller than 0.8 indicate strong synergism (dark gray); CIs smaller than 0.9 indicate synergism (gray); additivity (between 0.9 and 1.1) (light gray); or antagonism (more than 1.1) (white). Equipotent doses (50:50 cytotoxic ratio) of VPA and CDDP agents plus indicated doses of CX were evaluated after 144 h with a simultaneous (VPA + CDDP/CX) or sequential exposure with 24h delay to either drug (VPA→CDDP/CX; CDDP/CX→VPA), as described in section “Materials and Methods.” DRI values (mean ± SD from at least three separate experiments performed in quadruplicates) represent the order of magnitude (fold) of dose reduction obtained for IC_50_ (DRI_50_) in combination setting compared with each drug alone. Abbreviations: VPA (valproic acid); CDDP (Cisplatin); CX (Cetuximab).*

The synergistic antitumor interaction was confirmed by the DRIs evaluation. The order of magnitude (fold) of dose reduction obtained for the IC_50_ (DRI_50_) in combination *vs*. single drug treatments ranged, among the two cell lines tested, from about 1.37- up to 6.3-fold for CDDP/CX combination, considered as single treatment, and from about 1.95- up to 16-fold for VPA. In BJ-hTERT cells the combination did not results in any dose reduction in agreement with the absence of any synergistic interaction among the drugs tested (Table 1).

The synergistic antiproliferative effect induced by the triple VPA/CDDP/CX combination correlates in Cal27 cells with a significant synergistic induction of apoptosis compared to VPA or CDDP/CX alone, as evaluated by caspase 3/7 activity assay (Figure 1B) after 24 h at IC_50_^96h^ of each drug. Conversely, in FaDu cells, that resulted more sensitive to all the drugs tested we did not see any improvement in the early induction of apoptosis with the combination *vs*. single agents (Figure 1C). Moreover, in ZA and SCC9 cells, we confirmed the potentiation of the proapoptotic effect in the triple combination compared to VPA or CDDP/CX alone (Supplementary Figure S2). The combined antitumor effect was also demonstrated in Cal27 cells by a colony formation assay (Supplementary Figure S3).

**FIGURE 2:**
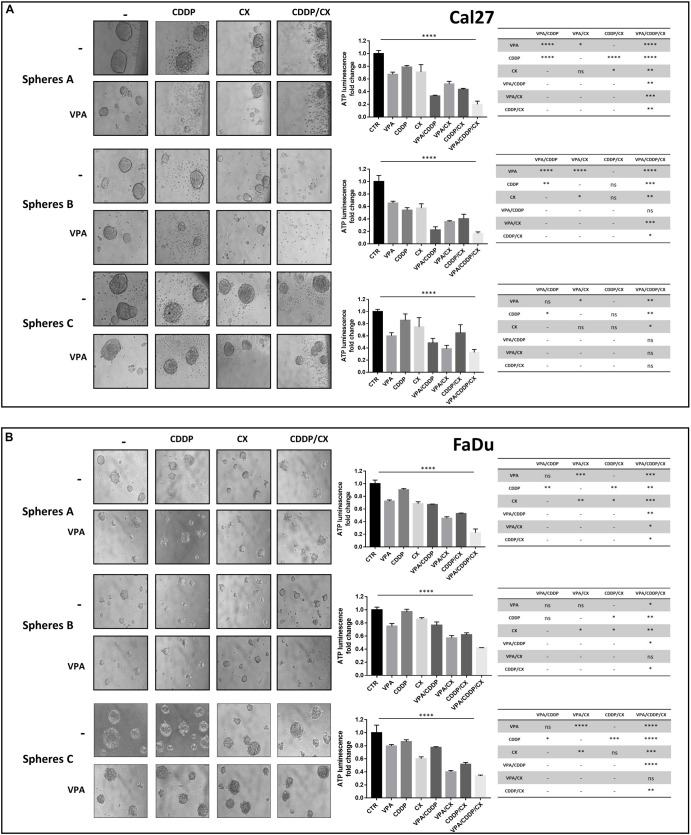
VPA in combination with CDDP/CX inhibits HNSCC spheroid formation and growth. Cal27 **(A)** and FaDu **(B)** cells (40,000/mL) were seeded in a sphere medium in a low attachment 96 multiwell as indicated in section “Materials and Methods.” *Spheres A*: cells were seeded and concomitantly treated with VPA and CDDP/CX at the indicated doses for 72 h. *Spheres B*: cells were grown for 72 h, then disaggregated and plated again in the presence of drugs for 72 h. *Spheres C*: spheres allowed to grow for 72 h and then treated for 72 h. Images were captured with 20× objective on a light microscope. Spheroids viability was assessed by luminescence assay. Statistical analysis: one-way ANOVA, ^****^p ≤ 0.0001, tables on the right shown values of unpaired *t*-test for each point. ^∗^*p* ≤ 0.05, ^∗∗^*p* ≤ 0.005, ^∗∗∗^*p* ≤ 0.001, ^****^p ≤ 0.0001.

Next, to better recapitulate tumor growth complexity, we tested VPA, CDDP, and CX as single agents and in combination, on Cal27 and FaDu cells 3D-self-assembled spheroids (Figures 2A,B). Notably, tumor-derived spheroids represent surrogate systems to evaluate our combined approach on cancer stem cells (CSCs) features. We used different models of spheroids in order to highlight different effects: (a) by evaluating treatments on 1^st^ generation sphere formation (cells plated in low-attached plate in sphere medium and concomitantly treated), we investigated the capacity of treatment to prevent/reduce tumor formation (*spheres A*); (b) by treating 2^nd^ generation sphere formation (cells were grown for 72 h, then disaggregated and plated again in the presence of drugs), we evaluated the impact of treatment to prevent/reduce more aggressive tumors (i.e., enriched in cells with extensive self-renewal capacity such as CSCs) (*spheres B*); (c) by treating formed-spheres (spheres allowed to grow for 72 h and then treated), we evaluated the capacity of treatment to induce tumor regression (*spheres C*). Our results showed that, in both Cal27 and FaDu cells, VPA/CDDP/CX combination strongly inhibits spheroid formation compared to single agents (Figures 2A,B). Notably, doublet combinations, using VPA either with CDDP or with CX alone, also demonstrated a potentiation of the antitumor activity compared with single agent treatments.

The impact of treatments on formed-sphere regression (*sphere C*) was less evident and different in the two cell lines. In FaDu cells that are sensitive to both CDDP and CX we observed a significant effect induced by either doublets VPA/CX and CDDP/CX or the triple combination *vs*. single agents or untreated controls. Interestingly, in Cal27, intrinsically resistant to both CDDP and CX, we observed, although not always statistically significant, a clear impact of VPA as single agent or in combination treatments *vs*. controls or other treatments (Figure 2A).

**FIGURE 3:**
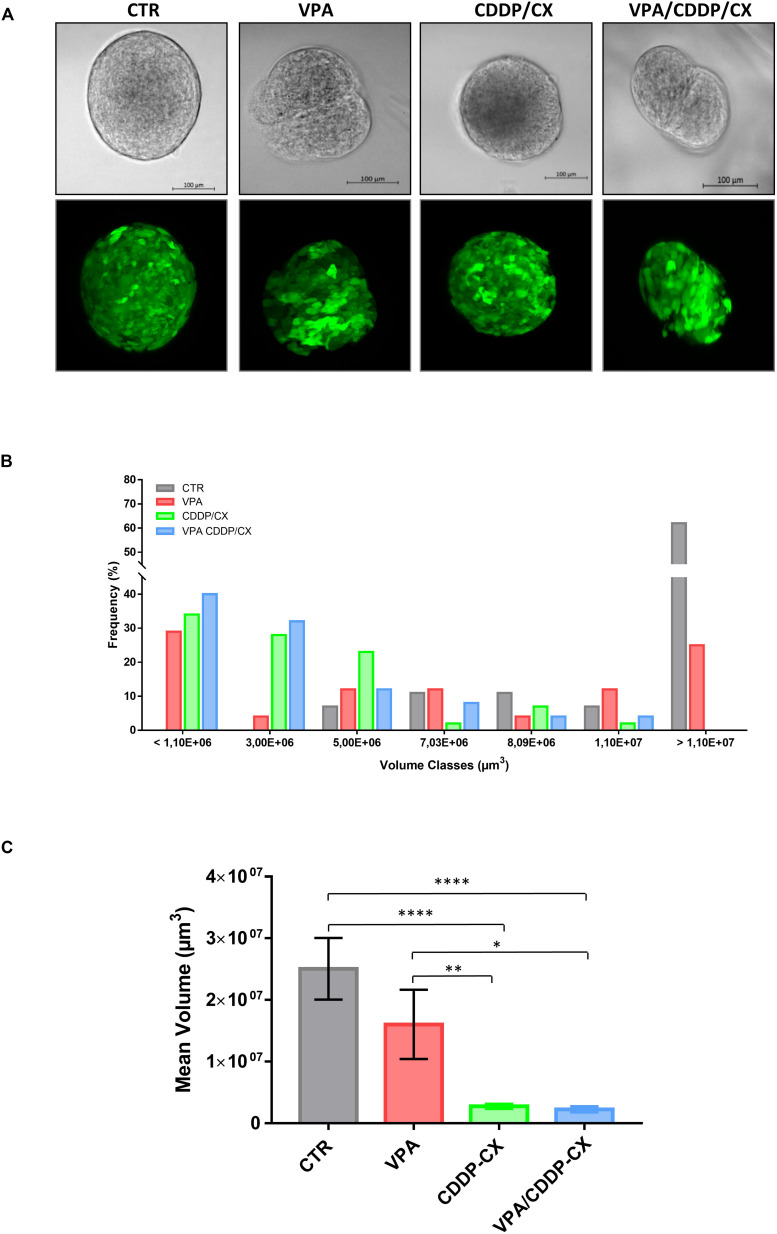
VPA in combination with CDDP/CX tackles self-renewal capacity of HNSCC cells grown as spheroids. Cal27-GFP^+^/Luc^+^ cells (40,000 cells/mL) were seeded in a sphere medium in a low attachment 96 multiwell to form spheroids: cells seeded and concomitantly untreated or treated with VPA and/or CCDP/CX at the respective IC_50_^96h^, then survived spheroids were disaggregated and plated again to form 2^nd^ generation spheroids in the absence of treatment. Spheroid growth was evaluated after 96h by using Zeiss Z1 light sheet fluorescence microscope. **(A)** Brightfield images (upper row) and maximum projection intensity fluorescence images obtained from each corresponding z-stack (lower row) are reported from representative spheroids. **(B)** Values expressed the percent of volume classes frequency distribution of the spheroids based on size (μm^3^), for untreated and treated groups (a minimum of 24 up to a maximum of 38 spheroids were evaluated for each group). **(C)** Values are the mean volumes ± SEM considering all spheroids examined and reported in panel **(B)**. Statistical analysis: unpaired *t*-test, ^∗^*p* ≤ 0.05, ^∗∗^*p* ≤ 0.005, ^****^p ≤ 0.0001.

Next, in order to further characterize the impact of the treatments on tumor spheroids, we took advantage of Cal27-GFP^+^/Luc^+^ cells, generated in our laboratory and previously described (Piro et al., 2019). In detail, to evaluate the potential of combination treatment to target more precisely the self-renewal capacity of CSCs, we pretreated the cells during the 1^st^ generation of spheroids formation and then surviving spheroids were disaggregated and plated again to form 2^nd^ generation spheroids without additional treatment. We visualized spheroids using a light sheet fluorescence microscope and showed the impact of treatments on the size and compactness of single spheroids (Figures 3A,B and Supplementary Videos 1–4). We demonstrated an enrichment of surviving small size cell spheroids in VPA, and particularly in CDDP/CX or triple combination pretreated cells, compared to controls, confirming the capacity of treatments to inhibit CSC self-renewal and thereby tumor spheroid growth (Figure 3B). Indeed, considering all visualized and measured spheroids, the mean volume was significantly reduced by both CDDP/CX and triple VPA/CDDP/CX combinations (Figure 3C). Notably, 3D visualization of the spheroids clearly highlighted the strong impact of all treatments, particularly of the triple combination, on cell density and 3D structure of single spheroids (Supplementary Videos 1–4).

Overall, these data suggest a synergistic antitumor interaction between VPA and CDDP/CX combination that occur both in adherent condition and in self-assembled spheroids, thereby suggesting the ability of the combined approach to affect also the cancer stem cells compartment.

**FIGURE 4:**
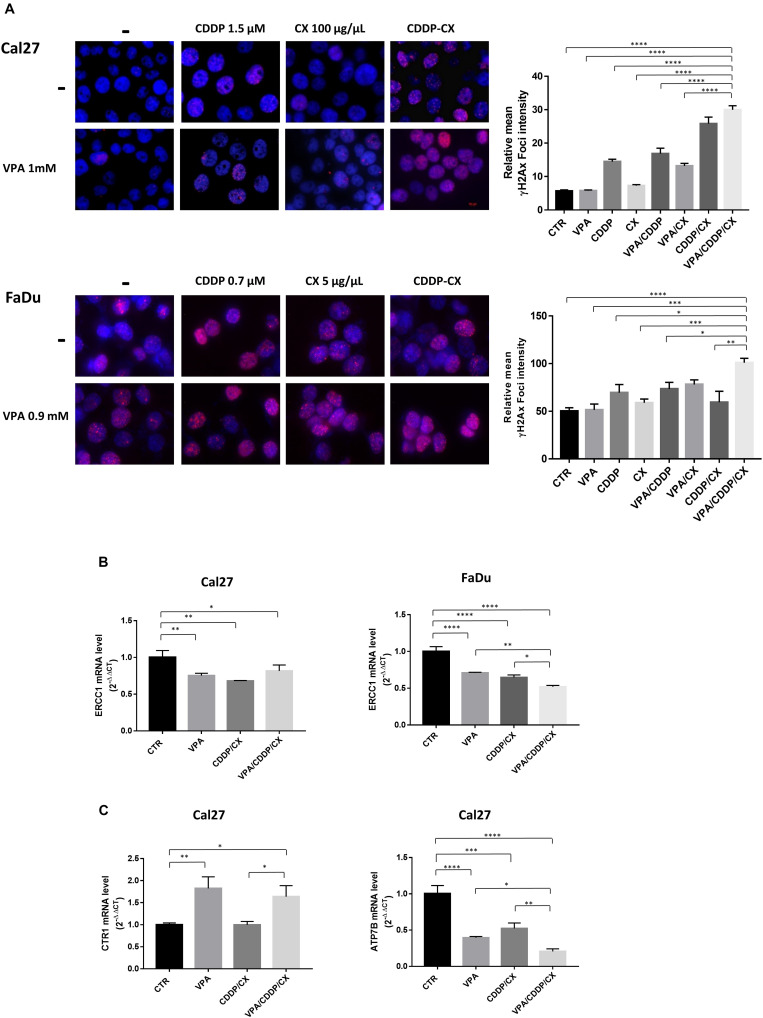
VPA in combination with CDDP/CX induces early DNA damage response. **(A)** Representative images of γH2AX foci evaluated by fluorescence confocal microscopy in Cal27 and FaDu cells untreated or treated as indicated for 24 h. Cells were stained with anti-γH2AX antibody (Alex Fluor594) and DAPI for nuclei detection (blue). Graphs on the right: foci quantification was performed by ImageJ analysis. **(B)** ERCC1 mRNA expression was evaluated by RT-PCR in Cal27 (left) and FaDu cells (right), after 24 h of cell culture in absence or presence of VPA and/or CDDP/CX at IC_50_^96h^ doses. **(C)** CTR1 (left) and ATP7B (right) mRNA expression was evaluated by RT-PCR in Cal27 cells. Statistical analysis: Tukey’s multiple comparisons test, ^∗^*p* ≤ 0.05, ^∗∗^*p* ≤ 0.005, ^∗∗∗^*p* ≤ 0.001, ^****^p ≤ 0.0001.

Cisplatin kills cancer cells by damaging their DNA. Therefore, we next measured DNA damage induction upon different treatments, by evaluating γH2AX nuclear foci formation in both Cal27 and FaDu cells (Figure 4A). We demonstrated that CDDP/CX treatment increased the number of γH2AX foci compared to single agent treatments and that this effect was clearly amplified by VPA, confirming the synergistic antitumor interactions demonstrated previously. Consistently, western blot analysis confirmed the increase of γH2AX expression upon triple combination treatment, also in two additional HNSCC cells, ZA and SCC9 (Supplementary Figure S4).

Mechanistically, the increased DNA damage observed in combination treatments could be related with the modulation of DNA repair mechanisms induced by HDACi, as previously reported (Terranova-Barberio et al., 2017). Indeed, we observed in both Cal27 and FaDu cells the significant mRNA reduction of ERCC1, a molecule playing an essential role in the removal of DNA intra-strand crosslinks by nucleotide excision repair, induced after 24 h by VPA as well as by CDDP/CX (IC_50_^96h^) and maintained or further increased in triple combination (Figure 4B). Moreover, in Cal27 cells, that express very low levels of CTR1 compared to FaDu cells, we also observed statistically significant mRNA upregulation of CTR1, induced by VPA as single agent or in triple combination (Figure 4C), confirming our previous observation obtained using the HDACi vorinostat (Piro et al., 2019). Notably, this effect is paralleled by the mRNA reduction of the ATPase ATP7B, which is involved in CDDP detoxification (Petruzzelli and Polishchuk, 2019), mediated by VPA and CDDP/CX alone, and further significantly reduced by triple combination (Figure 4C).

In summary VPA, potentiate the antitumor effect of CDDP/CX combination by increasing DNA damage effect impairing DNA repair, *via* ERCC1 downregulation, as well as facilitating the uptake of CDDP and preventing its efflux *via* CTR1 upregulation and ATP7B downregulation, respectively.

### VPA Inhibits EGFR Activation and CDDP/CX-Induced EGFR Nuclear Translocation

We have recently reported that the HDACi vorinostat was able to inhibit EGFR phosphorylation and nuclear translocation induced by CDDP plus 5FU combination treatment in HNSCC cells, thereby preventing a mechanism of chemo-resistance and potentiating the antitumor effect (Piro et al., 2019). Interestingly, EGFR nuclear localization was described as a specific mechanism of resistance to both CDDP and CX (Li et al., 2009; Han and Lo, 2012; Brand et al., 2014; Galluzzi et al., 2014).

Therefore, we next explored the putative role of EGFR modulation in the synergistic antitumor interaction between VPA and CDDP/CX. First of all, we demonstrated that low doses of HDACi VPA (0.5–1 mM) were able to downmodulate EGFR expression as well as the activation of the most important protein kinases downstream the EGFR-mediated signaling, such as AKT and MAPK, in both Cal27 and FaDu cells (Supplementary Figure S5A). Even more importantly, we demonstrated that VPA further downregulates these signals when combined with CDDP/CX (Supplementary Figure S5B).

**FIGURE 5:**
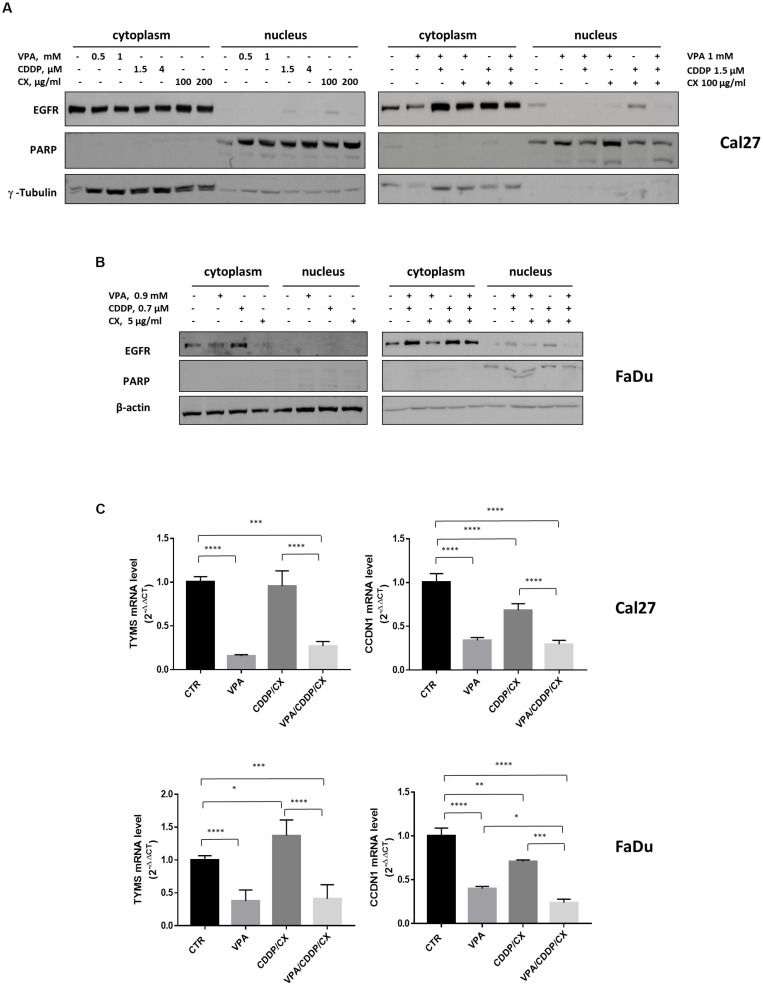
VPA reverts EGFR nuclear translocation induced by CDDP/CX. Western blotting analysis of nuclear and cytoplasmic EGFR expression in Cal27 **(A)** and FaDu **(B)** cells untreated or treated for 24h with VPA, CDDP and/or CX at the indicated doses. γ-tubulin and β-actin were used as loading control for cytoplasmic fraction and PARP was used as loading control for nuclear fraction. **(C)** TYMS and CCND1 mRNA expression was evaluated by RT-PCR in Cal 27 and FaDu cells untreated or treated for 24h with VPA, CCDP and/or CX at IC_50_^96h^ doses. Statistical analysis: Tukey’s multiple comparisons test, ^∗^*p* ≤ 0.05, ^∗∗^*p* ≤ 0.005, ^∗∗∗^*p* ≤ 0.001, ^****^p ≤ 0.0001.

Furthermore, we showed that either CX, or CDDP, as single agents, particularly in resistant Cal27 cells, and/or in combination in both Cal27 and FaDu cells, induced EGFR nuclear translocation and that VPA was able to revert this effect (Figures 5A,B). PARP expression ensured nuclear/cytoplasmic fractions separations and also demonstrated and increased cleavage in Cal27 cells in combination treatment, confirming the induction of apoptosis.

We have previously generated a genes signature induced by EGFR nuclear non-canonical activity, including two genes, TYMS and CCND1, upregulated by 5FU/CDDP chemotherapy in tumor cells (Piro et al., 2019). Thus, we evaluated the expression of TYMS and CCND1 transcripts in both Cal27 and FaDu cells untreated or treated with VPA, CDDP/CX, or the triple combination. As showed in Figure 5C, we demonstrated that VPA alone or in combination with CDDP/CX, strongly reduced TYMS and CCND1 mRNA levels in both cell lines.

### VPA in Combination With CDDP/CX Inhibits HNSCC Xenograft Tumor Growth

To assess whether the synergistic antitumor effects demonstrated *in vitro* could be confirmed *in vivo*, we explored the combination between VPA and CDDP/CX in Cal27 cells xenograft model in athymic mice.

**FIGURE 6:**
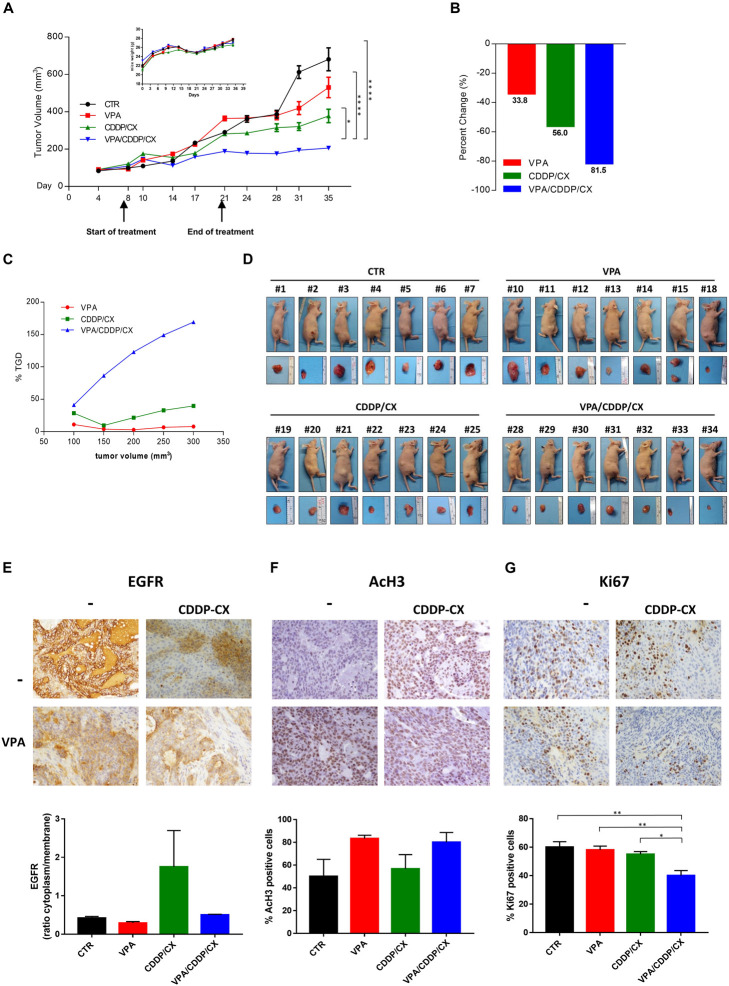
VPA and CDDP/CX completely block HNSCC xenograft tumor growth. Cal27 cells (6 × 10^6^) were s.c. injected into athymic mice as described in the section “Materials and methods.” When established tumors were palpable, mice were treated with VPA (200 mg/kg i.p. 5 days/week for 2 weeks), CDDP-CX (1 mg/kg i.p. and CX 1 mg/kg i.p. 3 days/week for 2 weeks), the three drugs in combination, or their vehicles. **(A)** Relative tumor volume curves for Cal27 xenografts; mean ± SD tumor volume measured at pre-specified time points (*n* = 7). Statistical analysis: Tukey’s multiple comparisons test, ^∗^*p* ≤ 0.05, ^****^p ≤ 0.0001. Inset, body weight measured three times/week. **(B)** Tumor volume averages from each group at day 8 and day 35 were compared and presented as percentages of vehicle. **(C)** Tumor growth delay, determined as%TGD = [(T – C)/C] × 100, where T and C are the mean times expressed in days for the treated or control groups, respectively, to reach a defined tumor volume (see Materials and Methods). **(D)** Photographs of sacrificed mice groups and of their excised tumors at the end of the *in vivo* experiment. **(E–G)** Pharmacodynamics markers on xenograft tumors. EGFR **(E)** and AcH3 **(F)** expression from tumors collected at the end of the treatment (day 21) and Ki67 **(G)** expression from tumors collected at the end of the experiment (day 35) were determined by IHC on FFPE tumor tissues using specific antibodies. Images were captured with 40× objective on a light microscope. Stained sections were scored semi-quantitatively for the percentage of positive cells and/or localization: EGFR intracellular/membrane ratio **(E)**, percent of nuclear AcH3 **(F)**, and Ki67 **(G)** positive cells, were reported. Statistical analysis: Tukey’s multiple comparisons test, ^∗^*p* ≤ 0.05, ^∗∗^*p* ≤ 0.005, ^****^p ≤ 0.0001.

Specifically, 36 engrafted mice were randomly assigned to receive subtherapeutic doses of VPA (200 mg/kgi.p.; 5 days/week for 2 weeks), CDDP/CX (CDDP at 1 mg/kg i.p. and CX at 1 mg/kg i.p.; 3 days/week for 2 weeks), the three drugs in combination, or their vehicles. As shown in Figure 6A, the triple combination treatment almost completely block tumor growth compared with controls or VPA and CDDP/CX treatments alone. In details, 35 days after cell injection, VPA/CDDP/CX treatment induced a significant inhibition of tumor growth compared with that in the untreated (*p* < 0.0001) or CDDP/CX (*p* = 0.0170) treated group (Figure 6A). The maintenance of body weight (inset in Figure 6A) and the absence of other acute or delayed toxicity signs indicated a well tolerability of this drugs combination.

Moreover, by calculating the percent change in tumor volume from the time of initial treatment (day 8) to the end of the study (day 35), we demonstrated that VPA, CDDP/CX, and triple combination treatment reduced the tumor burden by 33.8, 56, and 81.5%, respectively (Figure 6B).

Furthermore, we confirmed the synergistic antitumor interaction of VPA plus CDDP/CX *in vivo* by evaluating the TGD, demonstrating that in the mice treated with triple combination the resultant TGD reached a peak of more than 150%, indicating that the mean rate of tumor growth in the control was more than threefold higher (Figure 6C).

At day 21, which represents the end of the treatment, we sacrificed two mice per group (each with two tumors, one on each flank) to perform a pharmacodynamics analysis on tumor samples, while 2 weeks after the end of treatment (day 35) all the remaining mice were sacrificed and tumor specimens also collected (Figure 6D). In detail, we evaluated the expression and localization of EGFR by IHC analysis on FFPE tumor samples derived from mice sacrificed at the end of treatment (day 21). The percentage of EGFR expression was scored (Figure 6E) and interestingly, CDDP/CX combination induces an increase of EGFR, prevented by concomitant VPA treatment, suggesting also *in vivo* in xenograft tumors the occurrence of a putative non-canonical EGFR pathway induced by chemotherapy and reversed by VPA. At the same time point, an increase of AcH3 evaluated by IHC was demonstrated in tumor samples from both VPA and VPA plus CDDP/CX treated mice compared with controls and CDDP/CX groups, confirming the HDACi activity of VPA (Figure 6F). Finally, as shown by Ki67 staining on tumor samples explanted at day 35, the triple combination clearly affected tumor proliferation activity compared to untreated or single treatments mice (Figure 6G).

**FIGURE 7:**
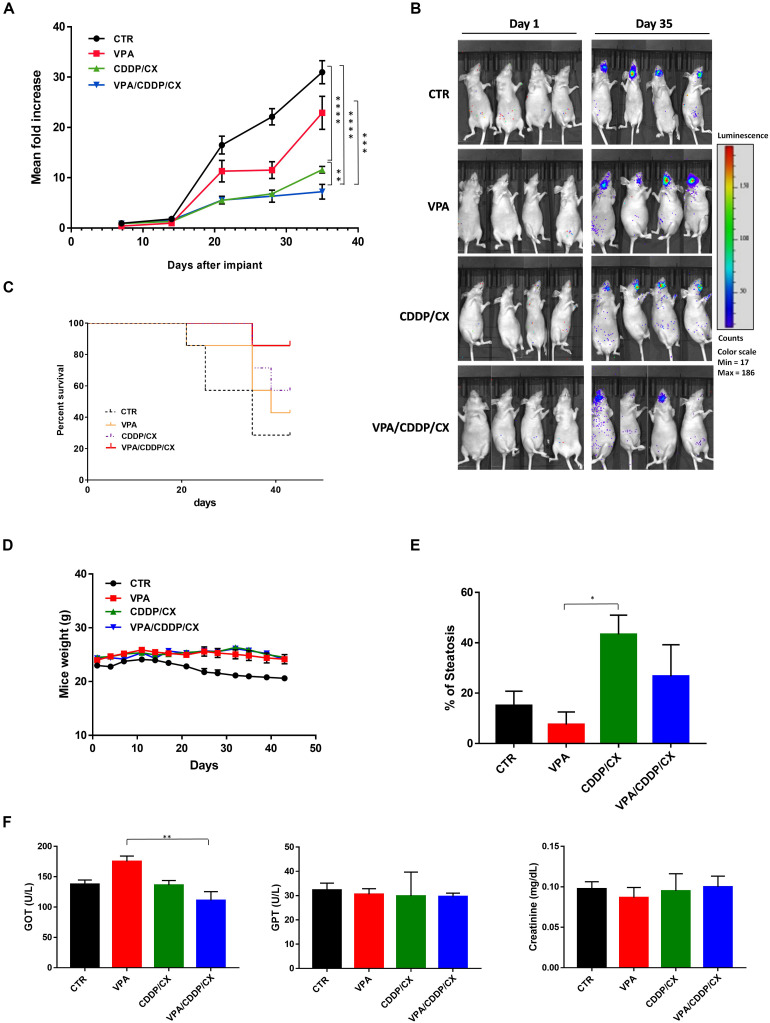
VPA and CDDP-CX effects on HNSCC orthotopic xenograft tumor growth. GFP^+^/luc^+^-transfected Cal27 cells (6 × 10^4^) were injected directly into the anterior tongue of athymic mice as described in the Materials and Methods. Four days after injection, mice were treated with VPA (200 mg/kg i.p. 5 days/week for 3 weeks), CDDP-CX (1 mg/kg i.p. and CX 1 mg/kg i.p. 3 days/week for 3 weeks), the three drugs in combination, or their vehicles. **(A)** Tumor volume was quantified as the sum of all detected photons within the region of the tumor/s. The mean of each measurement was compared with the mean at day 1. Relative fold increase values were reported in the graph as mean ± SEM. Statistical analysis: Welch’s *t*-test, ^∗∗^*p* ≤ 0.005, ^∗∗∗^*p* ≤ 0.001, ^****^p ≤ 0.0001. **(B)** Representative *in vivo* images (IVIS) of luminescence shown in the tongue of live mice at day 1 and day 35 after injection. Rainbow images show the relative levels of luminescence ranging from low (blue), to medium (green), to high (yellow/red). Luminescence levels (photons/s) of 4 out of 10 mice/groups. **(C)** Kaplan–Meier survival curves. Mice were sacrificed when the photons total count was higher than 3,000 or mice loss 10% of body weight during the study period. **(D)** Body weight of mice, measured three times/week, was reported as mean ± SEM. **(E)** Percentage of hepatic steatosis was measured on H&E-stained paraffin section of the liver from mice sacrificed at the end of treatment. **(F)** GOT, GPT, and creatinine serum levels in mice sacrificed at the end of treatment. Statistical analysis: Tukey’s multiple comparisons test, ^∗^*p* ≤ 0.05, ^∗∗^*p* ≤ 0.005.

Finally, we confirmed the synergistic interaction of the proposed combination in an orthotopic xenograft *in vivo* model, which better recapitulate the HNSCC tumor microenvironment, by taking advantage of Cal27-GFP^+^/Luc^+^ cells injected into the tongue of mice. Four days after implantation the mice were randomly assigned to receive subtherapeutic doses of VPA (200 mg/kgi.p.; 5 days/week for 3 weeks), CDDP/CX (CDDP at 1 mg/kg i.p. and CX at 1 mg/kg i.p.; 3 days/week for 3 weeks), the three drugs in combination, or their vehicles (Figure 7). Tumor growth was monitored two times/week by photon intensity and, for each group, the means of the measurements were compared with the mean at day 1 (Figure 7A). The major tumor growth inhibition was observed in the VPA/CDDP/CX combination at day 35 (Figures 7A,B). Consequently, we also obtained significant improvement of mice survival in triple combination group, as demonstrated by Kaplan-Meier plot reported in Figure 7C. We also confirmed the absence of major toxicity in treated mice by maintenance of body weight (Figure 7D), histology examinations of the liver (Figure 7E), and biochemical examinations on mice blood of GOT, GPT, and creatinine (Figure 7F). In details, three mice from each group were sacrificed at the end of treatment (day 21), and tissues and blood samples were collected in order to perform pharmacokinetic analysis. Although CDDP/CX treatment increased liver steatosis, triple combination did not increase this effect (Figure 7E). Furthermore, no significant changes of the biochemical parameters (GOT, GPT, and creatinine) were reported in serum of untreated mice compared with treated groups, with the exception of a slight increase of GOT induced by VPA, confirming again that the triple combination did not increase significantly the liver toxicity of the single treatments (Figure 7F).

## Discussion

Despite the many studies that were devoted to defining the genetic and molecular mechanisms of HNSCC initiation and progression, they did not result in the development of novel therapeutic approaches. Similarly, rather than molecular characterization, Tumor, Nodes, Metastasis (TNM) classification of malignant tumors and other clinical factors, with the exception of HPV status, are still the major drivers for HNSCC patient’s prognosis. Indeed, excluding the subgroup of genetically distinct HPV positive tumors with a favorable prognosis, the treatment approach for R/M HNSCC patients require novel strategies, including optimization of current available effective therapies.

We addressed, at least in part, these challenges by suggesting a novel therapeutic approach based on the use of the antiepileptic VPA, a generic very safe well-known drug with HDACi activity, in combination with the CDDP/CX doublet, which still represent and important effective therapeutic option for HSNCC.

Toxicity and therapeutic resistance are among the major contributors to therapeutic failure to both CDDP and CX in HNSCC (Saba et al., 2017; Lopez-Verdin et al., 2018).

Although several reports showed a synergistic antitumor effect by combining CDDP with an HDACi, including VPA, via DNA-damage induction and DNA repair inhibition (Diyabalanage et al., 2013; Huang et al., 2018; Suraweera et al., 2018; Piro et al., 2019), to our knowledge this study is the only one demonstrating an effective combined approach with both CDDP and CX, both *in vitro* and *in vivo*. We also demonstrated for the first time a synergistic interaction by combining VPA with CX as single agent.

In detail, in our study, we reported several evidences suggesting that VPA can tackle both CDDP and CX resistance through several mechanisms: (1) by specifically targeting CSCs compartment; (2) by decreasing DNA repair mechanisms and concomitantly increasing CDDP concentrations within tumor cells regulating influx and efflux mechanisms; and (3) by reverting EGFR activation and nuclear translocation, thereby impairing the activation of survival pathways and DNA repair mechanisms.

Previous findings demonstrated the presence of CSCs subpopulation in HNSCC responsible for tumor initiation progression and drug resistance (Chen and Wang, 2019). Moreover, it was reported that CDDP induced the promotion and the maintenance of CSCs subpopulation leading to drug resistance (Thakur and Ray, 2017). We have recently demonstrated by a bioinformatics analysis that selected pathways described as hyperactivated in CSCs are altered in several solid tumors, including HNSCC, and enriched in patients with bad prognosis (Roca et al., 2019). Notably, HDACi appears particularly suitable in targeting such pathways (Roca et al., 2019).

Indeed, here we demonstrated in spheroids culture, which are particularly enriched in CSCs, that VPA alone and even more in combination with CCDP and/or with CX, strongly inhibits HNSCC spheroids generation, compared with non-efficacious CDDP or CX single agents, or even CDDP/CX.

Interestingly, it was proposed that CSCs chemo-resistance is associated with their enhanced DNA damage response (Abad et al., 2020). HDACs participate in the DNA damage response particularly in the early event (Sun et al., 2019) and even more interestingly CDDP treatment induced HDACs in tumor cells, thus contributing to resistance and to the expansion of CSCs subpopulation (Wang et al., 2017). Indeed, HDACi treatment impairs DNA repair mechanisms thus potentiating CDDP antitumor effect (Gomez-Gonzalez et al., 2020). Previously we have reported that VPA was able to prolong and further increase the DNA damage induced by fluoropyrimidines plus radiotherapy in colorectal cancer cells (Terranova-Barberio et al., 2017) or by CDDP/5FU in squamous cancer cell lines (Piro et al., 2019). Here we reported a precocious induction of DNA damage in combination setting, as shown by increased γH2AX foci formation.

This effect, is most likely due to a decrease of repair rate of DSB, depending on downmodulation of ERCC1, which has an essential role in the removal of DNA intra-strand crosslinks by nucleotide excision repair, thus resolving platinum-DNA damage (Galluzzi et al., 2014), induced by VPA alone or in combined treatment. In parallel, the enhanced DNA damage obtained in combination setting is, most likely, also due to the increased concentrations of CDDP within the CDDP-resistant Cal27 cells, *via* VPA-induced upregulation of CDDP-influx channel CTR1 (Galluzzi et al., 2014) and downregulation of the ATPase ATP7B involved in CDDP detoxification (Galluzzi et al., 2014; Petruzzelli and Polishchuk, 2019). Notably, the increase of ERCC1 expression or of ATP7B and the loss of CTR1 have been all consistently reported as mechanisms of platinum resistance as well as predictors of poor response to platinum-based chemotherapy in cancer patients, including HNSCC (Bisof et al., 2016; Sun et al., 2017; Petruzzelli and Polishchuk, 2019). ERCC1 downregulation by HDACi was previously reported (To et al., 2017); however, to our knowledge, this is the first report showing the effect of VPA. We have recently demonstrated that the HDACi vorinostat enhanced the platinum intracellular concentration in squamous cancer cells *via* the upregulation of CTR1 and, in this way, enhanced, *in vitro* and *in vivo*, the antitumor effect of CDDP/5FU combination (Piro et al., 2019). Here we confirmed this effect with VPA, demonstrating a significant induction of CTR1 transcripts in CDDP intrinsically resistant Cal27 cells that express very low basal levels of CTR1 protein compared with CDDP-sensitive FaDu cells. However, we also showed, for the first time, a parallel VPA-induced downregulation of ATP7B, an original observation of critical interest in our opinion. Indeed, increased expression of the Golgi-localized Cu-transporting ATPases, ATP7A and ATP7B, have been associated to tumor aggressiveness as well as chemo-resistance. In details, preclinical studies in platinum-resistant cell lines, as well clinical observations, revealed that ATP7B expressing tumors had poor response to CDDP-based chemotherapy (Petruzzelli and Polishchuk, 2019). However, the exact mechanism of how ATP7A/B contribute to platinum detoxification and trafficking within the cells, is not completely defined, as well as it is not yet consolidated the correct strategy to pharmacological targeting this proteins (Petruzzelli and Polishchuk, 2019).

High levels of EGFR are often reported in HNSCC, and elevated expression of EGFR also enhances the proportion of CSCs subpopulation in HNSCC (Chen and Wang, 2019). However, anti-EGFR agent such as CX are not so efficacious in controlling HNSCC CSCs (Chen and Wang, 2019). Conversely, it is well-known that CDDP induced EGFR activation as a survival response (Benhar et al., 2002), and this represents the rationale for the clinical combination with CX. It was also reported that an EGFR downstream pathway such as PI3K/AKT signaling is activated by CDDP in resistant cells and that this contributes to promotion and maintenance of CSC subpopulation (Thakur and Ray, 2017). Furthermore, a non-canonical nuclear localization of EGFR, mediating transcription of DNA-repair and survival response genes, was reported as induced by DNA damage approach such as radiotherapy and CDDP, as well as by anti-EGFR agents including CX (Li et al., 2009; Han and Lo, 2012; Brand et al., 2014).

We have previously shown that the HDACi vorinostat, in combination with the EGFR-tyrosine kinase inhibitor gefitinib, induced synergistic antitumor interaction in preclinical models of HNSCC and non-small cell lung cancer with a mechanism based, among others, on the ability of vorinostat to modulate the expression and the activity of ErbB receptors (EGFR, ErbB2, and ErbB3) (Bruzzese et al., 2011; Leone et al., 2015; Citro et al., 2019; He et al., 2019). We also demonstrated that both vorinostat and VPA downregulate EGFR protein expression mainly by increasing protein degradation in HNSCC and non-small cell lung cancer cell lines and tumor primary cultures (Bruzzese et al., 2011; Ciardiello et al., 2016).

Finally, recent data from our group demonstrated that vorinostat is able to inhibit CDDP/5FU-induced phosphorylation and nuclear translocation of EGFR, an effect that contribute to the synergistic antitumor effect obtained in combination setting (Piro et al., 2019). In the present study, we confirmed that VPA downregulates EGFR expression and downstream main activating signaling molecules, such as MAPK and AKT, as single agents and synergistically in triple combination with CDDP/CX. However, more importantly we demonstrated, for the first time, that VPA is able to prevent EGFR nuclear translocation induced by either CDDP or CX alone and in combination. Notably, also *in vivo* experiments on HSNCC xenograft tumor samples we observed an increased intracellular EGFR localization upon CDDP/CX treatment that was prevented by concomitant treatment with VPA. Functionally these data were associated with the significant reduction, induced by VPA alone or in triple combination, of TYMS and CCND1 mRNA expression, both part of a genes signature induced by EGFR nuclear non-canonical activity that we have generated in a recent report (Piro et al., 2019). Notably, both TYMS and CCND1 were recently reported to be essential for the maintenance of CSCs subpopulation (Siddiqui et al., 2019; Zhang, 2020), and to be highly enriched in poor- compared with good-outcome HNSCC patients (Piro et al., 2019).

Overall, we demonstrated that by combining VPA with CDDP/CX we clearly potentiate the antitumor effect in HNSCC models *in vitro* and *in vivo*, in both heterotopic and orthotopic xenograft models, most likely by tackling several reported mechanisms of resistance for both CDDP and CX.

Remarkably, these mechanisms of resistance are validated therapeutic targets in HNSCC. For instance, a meta-analysis of more than 1,000 patients demonstrated that high ERCC1 expression was significantly related with shorter progression-free and overall survival in CDDP-treated HNSCC patients (Bisof et al., 2016). Moreover, automated quantitative assessment of nuclear and cytoplasmic EGFR IHC expression in 100 HNSCC specimens demonstrated, even within a multi-variate analysis, that, high tumor and nuclear EGFR expression were associated with higher local recurrence and inferior disease-free survival compared with low expressing tumors (Psyrri et al., 2005). Furthermore, direct targets of VPA HDACi activity, such as histone modification pattern like hypoacetylation of histone H3, as well as overexpression of different HDACs, were reported to be associated with progression and poor prognosis of HNSCC patients (Castilho et al., 2017). We should underline that the most significant results, including the *in vivo* study, were obtained using the Cal27 cell model, intrinsically resistant to CDDP and CX and molecularly distinct from more sensitive FaDu cells. Therefore, future challenges are related with the validation of predictive biomarkers, based on the mechanisms described above, able to select patients potentially more prone to respond to the triple combination treatment.

Few clinical trials investigating the potential of HDACi in HNSCC have been reported or are currently ongoing, with limited activity as single agents, but with promising results in combination therapy (Haigentz et al., 2012; Teknos et al., 2019). In detail, a recently concluded phase I trial reported the feasibility and the efficacy of vorinostat in combination with concurrent standard chemo-radiation in HNSCC patients, suggesting further investigations in phase II/III trials for this combination (Teknos et al., 2019).

We reported a synergistic antitumor interaction, both *in vitro* and *in vivo*, using dosages of VPA, CDDP, or CX consistent with those found in the serum of patients following treatment of these drugs (VPA range 0.3–1 mM; CDDP 1.9–8.2 μM; CX 49.4–155.8 μg/mL) (Panteix et al., 2002; Arce et al., 2006; Tan et al., 2006). Moreover, the synergistic interactions not dependent on the treatment schedule used is an observation that could be clinically relevant because a less stringent condition of drug administration and would make this combination easily adaptable for clinical application. It is also important to underline that VPA exerted its molecular effects at dosages reported in the plasma of patients treated with the safe antiepileptic regimen. Moreover, the only dose limiting toxicities reported for VPA are neovestibular symptoms, fatigue, and somnolence (Avallone et al., 2014), thus suggesting that the combination treatment could be potentially clinically explored without further exacerbating side effects or impairing the quality of life. Of note, the lack of synergistic interaction observed in normal human fibroblasts suggested a selective action on tumor cells and thus, again a good therapeutic index of the combined approach. This latter observation was also confirmed *in vivo*, in both heterotopic and orthotopic xenograft models, by demonstrating synergistic antitumor effect of triple combination, resulting in significant improvement of survival, in the absence of increased toxic effects.

Anyhow, as VPA and CDDP/CX synergistically inhibited tumor growth we can reduce dosages of all the drugs in the clinical setting thereby eventually further reducing toxicities. Indeed, we reported significant DRIs values for all the drugs, indicating that besides the potentiation of CDDP/CX effect induced by VPA, conversely CDDP/CX enhanced VPA effect when in combination. This latter observation suggest a complex and efficacious synergistic mechanism of interactions and is in line with a recent report demonstrating that CDDP is able to enhance the anticancer effect of the HDACi entinostat (Huang et al., 2018). Thereby, we cannot exclude that additional mechanisms can be involved in the strong antitumor synergism we have observed VPA and CDDP/CX.

We have been demonstrated the feasibility of VPA used at standard anti-epileptic dosage in combinatory approach in ongoing clinical trials in cancer patients. The phase I trial of VPA in combination with capecitabine during pre-operative radiotherapy, in locally advanced rectal cancer patients, was recently concluded (Budillon et al., 2018), and we are enrolling patients in the phase II study. Moreover, a randomized phase II trial of bevacizumab plus fluoropyrimidines and oxaliplatin-based chemotherapy with or without VPA, in first-line RAS-mutated colorectal cancer patients, is currently enrolling patients (Revolution Trial, NCT04310176).

In the current study, we provided a rationale to clinically explore VPA in combination with CDDP and CX to overcome chemotherapy resistance and dose-limiting toxicity. Indeed based on our preliminary observations, the phase II V-CHANCE clinical trials is currently ongoing in our institute with the aim to demonstrate the feasibility and efficacy of VPA in combination with CDDP and CX in R/M HNSCC patients (Caponigro et al., 2016). This trial is the only one investigating VPA in HNSCC, and the first trial ever combining an HDACi with the anti-EGFR antibody CX. The planned correlative studies could add new insight in the mechanism of synergistic interaction between the tested drugs in cancer patients also confirming our preclinical findings. In detail, we could identify predictive biomarkers to identify chemo-resistant tumors with driver HDACi targetable chemo-escape pathways.

## Data Availability Statement

The raw data supporting the conclusions of this article will be made available by the authors, without undue reservation.

## Ethics Statement

The animal study was reviewed and approved by OPBA CROM Ist. Pascale, Napoli and Italian Minister of Health (Approval N.865/2015-PR).

## Author Contributions

FIa, AZ, ED, AB, FIo, and FC: conception and design. FIa, AZ, ED, MR, LG, RL, FB, CC, TM, CV, AL, GI, and SV: development of methodology. FIa, AZ, ED, MR, LG, RL, MM, CC, TM, CV, AL, GI, SV, and EC: acquisition of data (provided animals, acquired and managed patients, provided facilities, etc.). FIa, AZ, ED, MR, RL, FB, AL, and AB: analysis and interpretation of data (e.g., statistical analysis, biostatistics, and computational analysis). FIa, ED, LG, and AB: writing, review, and/or revision of the manuscript. FIa, AZ, ED, MR, LG, RL, FB, CC, TM, CV, and AL: administrative, technical, or material support (i.e., reporting or organizing data and constructing databases). All authors contributed to the article and approved the submitted version.

## Conflict of Interest

The authors declare that the research was conducted in the absence of any commercial or financial relationships that could be construed as a potential conflict of interest.

## Footnotes

1 GLOBOCAN. Global Cancer Statistics 2018: GLOBOCAN estimates of incidence and mortality worldwide for 36 cancers in 185 countries; 2018. Retrieved February 19, 2019, from http://gco.iarc.fr/today/fact-sheets-populations

2 National Comprehensive Cancer Network. NCCN Clinical Practice Guidelines in Oncology: Head and Neck Cancer from https://www.nccn.org/professionals/physician_gls/recently_updated.aspx

3 FDA approves pembrolizumab for first-line treatment of head and neck squamous cell carcinoma from https://www.fda.gov/drugs/resources-information-approved-drugs/fda-approves-pembrolizumab-first-line-treatment-head-and-neck-squamous-cell-carcinoma

4 https://clinicaltrials.gov/ct2/results?cond=cancer&term=valproic+acid&cntry=&state=&city=&dist=

5 Avallone et al.’s (2014) randomized phase 2 study of Valproic acid in combination with bevacizumab and Oxaliplatin/fLUoropyrimidine regimens in patients with ras-mutated metastatic cOlorectalcaNcer(REVOLUTION TRIAL, NCT04310176), https://clinicaltrials.gov/ct2/show/NCT04310176?term=revolution&recrs=ab&cond=Colo-rectal+Cancer&draw=2&rank=1
