# Supplementary material for: Valproic Acid Synergizes With Cisplatin and Cetuximab in vitro and in vivo in Head and Neck Cancer by Targeting the Mechanisms of Resistance
Source: Front Cell Dev Biol. 2020 Aug 17;8:732. doi: 10.3389/fcell.2020.00732 (PMC7461984; doi:10.3389/fcell.2020.00732)
Supplement: Supplementary file 5 [file Data_Sheet_1.pdf]

Supplementary Material

1 Supplementary materials

Reagents

Prymary antibodies for western blotting according to the manufacturer's protocol: phospho-Epidermal Growth Factor Receptor (p-EGFR)-Ab (Cell Signaling Technology, #3777), Epidermal Growth Factor Receptor (EGFR)-Ab (Cell Signaling Technology, #4267), phospho-p44/42 MAPK (pErk1/2)-Ab (Cell Signaling Technology, #4370), p44/42 MAPK (Erk1/2)-Ab (Cell Signaling Technology, #9102), phospho-Akt (pAKT)-Ab (Cell Signaling Technology, #4060), Akt-Ab (Cell Signaling Technology, #2920), β-Actin-Ab (Abcam, ab8227), poly-(ADPribose)-Polymerase (PARP)-Ab (BD Biosciences, cod. 556494), γ-Tubulin-Ab (Santa Cruz, cod. sc-7396), phospho-Histone H2AX (γH2AX)-Ab (Millipore, 05636), SLC31A1/CTR1-Ab (Novus Biological NB100-402). Secondary antibodies were purchased as follows: polyclonal goat anti-rabbit IgG (H+L)-HRP conjugate (Bio-Rad, #1706515), polyclonal rabbit anti-goat IgG-HRP conjugate (Santa Cruz, cod. sc-2768) and polyclonal goat anti-mouse IgG (H+L)-HRP conjugate (Bio-Rad, #1706516), Goat polyclonal Secondary Antibody to Mouse IgG - H&L - Alexa Fluor® 594 (Abcam, #ab150120).

Clonogenic assay

Single Cal27 cell suspensions were plated at 150–200 cells/well in 6 wells plate for clonogenic assay and treated or untreated with IC_{15} at 96h concentrations of VPA (0.3 mM) and CDDP (0.5 μM) and 100 μg/mL of CX. After 12 days, colonies were visualized by incubation with 0.5% crystal violet dissolved in 20% methanol for 30 min. Colonies were photographed, analyzed and the colonies aria was evaluated using image-Pro-Plus (Immagini and Computer snc).

Cytofluorimetric assay

EGFR expression was measured on both Cal27 and FaDu cells by flow cytometry using a FACS Calibur (Becton Dickinson, San Jose, CA, USA) staining cells with APC anti-human EGFR antibody (SONY, 2364525) according to the manufacturer's protocol. The data were analyzed using the Cell Quest Pro software package (Becton Dickinson, San Jose, CA, USA).

2 Supplementary Tables

Supplementary Table S1. Screening of HNSCC cell lines, antiproliferative effect of drugs alone.

Abbreviations: VPA: Valproic Acid; CDDP: Cisplatin; CX: cetuximab. Half maximal inhibitory concentration (IC_{50}) of VPA, CDDP and CX calculated after 96h of treatment in Cal27, FaDu, ZA, HOC313 and SCC9 cell lines (mean ± standard deviation from at least three separate experiments performed in quadruplicates). Cell growth assessment was done by sulforhodamine B colorimetric assay (see Materials and Methods).

**Cell Lines**	**VPA, mM**<br>**IC_{50} 96h ± SD**	**CDDP, µM**<br>**IC_{50} 96h ± SD**	**CX, µg/mL**<br>**IC_{50} 96h ± SD**
**Cal27**	1.24 ± 0.30	1.22 ± 0.25	> 250
**FaDu**	0.89 ± 0.12	0.67 ± 0.06	4.8 ± 5.7
**ZA**	3.39 ± 0.5	3.8 ± 0.4	> 100
**HOC313**	3.8 ± 0.14	8.3 ± 0.7	> 100
**SCC9**	4.85 ± 1.2	1.74 ± 0.22	> 100

Supplementary Table S2. Characteristics of HNSCC cell lines.

Abbreviations: mut: mutation; wt: wild type; Neg: negative; unk: unknown

**Cell line / origin**	**p53 status**	**RAS status**	**PI3K status**	**ErbBs expression**	**TERT promoter**	**HPV infection**	**Others mut**
**Cal27**<br>Tongue	Mut<br>p.H193L	Rs61764370<br>NRAS mut:<br>p.D92N<br>p.R68T	mut<br>PIK3CG<br>p.R178C	EGFR+++<br>HER2++<br>ErbB3++	wt	Neg	ABL1.<br>SMAD4.<br>CASP8.<br>TSHR.<br>APC<br>p16
**FaDu**<br>Pharynx	Mut<br>p.R248L	wt	wt	EGFR+<br>HER2+<br>ErbB3+	wt	Neg	SMAD4.<br>CXCR4<br>p16
**ZA**<br>cervical<br>lymph node<br>oral cavity	Mut	unk	wt	EGFR<br>amplification	unk	unk	unk
**HOC313**<br>lymph node<br>floor mouth	Mut	Mut<br>Kras cod12	wt	EGFR wt	unk	unk	CDKN2
SCC9<br>Tongue	Mut	unk	unk	unk	unk	unk	hom del<br>CDKN2<br>ALDH1L1<br>ALK

Supplementary Table S3. Antiproliferative effect induced by VPA/CDDP combination according to the different schedules of exposure in Cal27 and FaDu cell lines.

Cell Lines	Treatment	CI_{50} 96h ± *SD*	CI_{75} 96h ± *SD*	CI_{90} 96h ± *SD*	DRI_{50} 96h ± *SD*	
					VPA	CDDP
Cal27	VPA + CDDP	0.94 ± 0.06	0.76 ± 0.08	0.61 ± 0.10	1.97 ± 0.54	2.73 ± 0.78
	VPA → CDDP	0.86 ± 0.05	0.69 ± 0.01	0.57 ± 0.03	1.95 ± 0.22	2.93 ± 0.24
	CDDP → VPA	0.94 ± 0.08	0.77 ± 0.08	0.64 ± 0.11	1.43 ± 0.15	4.27 ± 0.37
FaDu	VPA + CDDP	0.92 ± 0.02	0.79 ± 0.04	0.69 ± 0.05	1.77 ± 0.36	3.31 ± 1.07
	VPA → CDDP	0.83 ± 0.08	0.65 ± 0.07	0.51 ± 0.05	3.18 ± 0.22	1.95 ± 0.25
	CDDP → VPA	0.92 ± 0.06	0.82 ± 0.02	0.77 ± 0.05	1.60 ± 0.04	4.08 ± 0.12

Synergistic interaction between VPA and CDDP evaluated in Cal27 and FaDu cells by calculation of combination index (CI) values when 50%, 75% and 90% of the cells had died (CI_{50}, CI_{75} and CI_{90}, respectively) according to CalcuSyn software after 96 hours of treatment (Mean ± SD from at least three separate experiments performed in quadruplicate). CIs smaller than 0.8 indicate strong synergism (dark grey); CIs smaller than 0.9 indicate synergism (gray); additivity (between 0.9 and 1.1) (light gray); or antagonism (more than 1.1) (white). Equipotent doses (50:50 cytotoxic ratio) of each of the two agents were evaluated after 96h with a simultaneous (VPA+CDDP) or sequential exposure with 24h delay to either drug (VPA→ CDDP; CDDP→VPA) as described in Materials and Methods. DRI values (mean ± SD from at least three separate experiments performed in quadruplicates) represents the order of magnitude (fold) of dose reduction obtained for IC_{50} (DRI_{50}) in combination setting compared with each drug alone.

Abbreviations: VPA (valproic acid); CDDP (Cisplatin).

3 Supplementary Figures

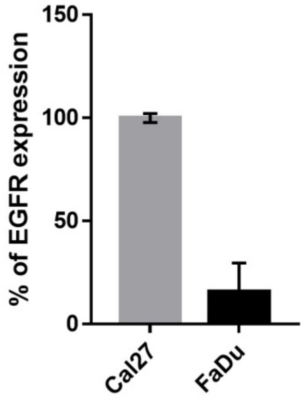

Supplementary Figure S1. Basal expression of EGFR in Cal27 and FaDu cells.

EGFR basal expression was determined by flow cytometry analysis in Cal27 and FaDu HNSCC cell lines. Cells were harvested after 24h and stained with APC anti-human EGFR antibody.

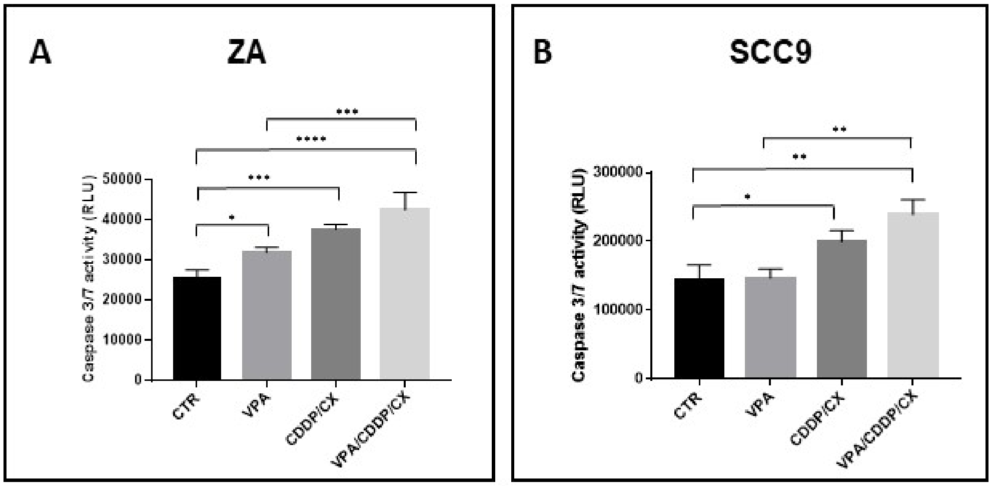

Supplementary Figure S2. Pro-apoptotic effects induced by VPA/CDDP/CX in HNSCC cells.

Caspase 3/7 activity was evaluated in ZA (**A**) and SCC9 (**B**) cells untreated or treated with VPA and/or CDDP/CX at IC_{50}^{96h} doses for 24h, by luminescence assay. Results are shown as mean +/- SD. (Statistical analysis: Tukey's multiple comparisons test, $^{*}p \leq 0.05$, $^{**}p \leq 0.005$, $^{***}p \leq 0.001$, $^{****}p \leq 0.0001$).

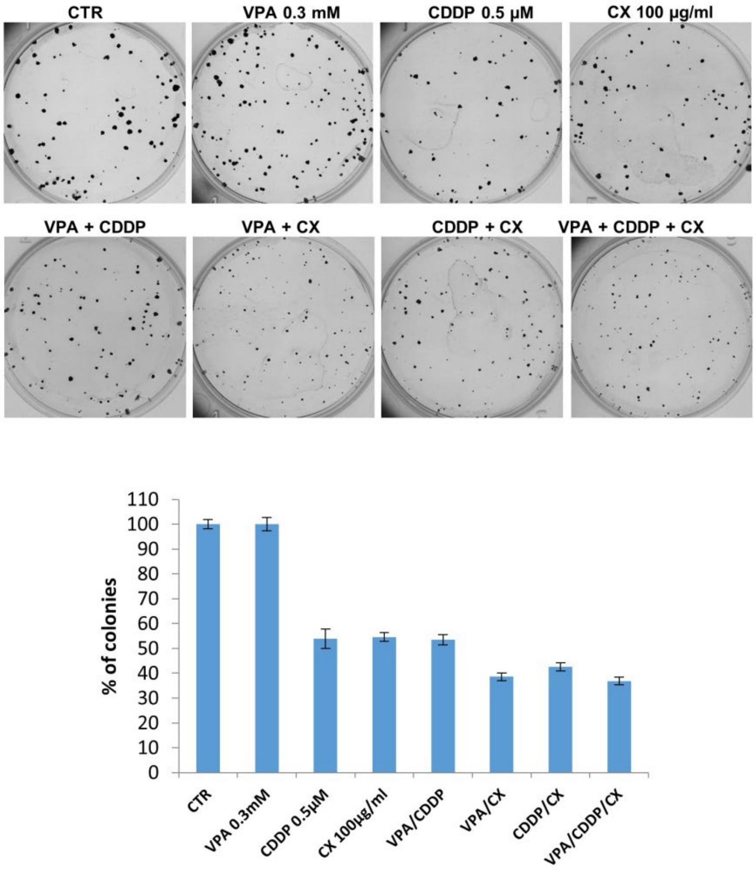

Supplementary Figure S3. Effects of VPA alone or in combination with CDDP/CX on colony formation.

Cal27 cells were untreated or treated as indicated and collected after 12 days. A photograph of one well in a representative experiment is shown for each treatment; bar graphs show the percentage of inhibition of colony formation by drug combination treatment (mean ± SD of 3 or more separate experiments each one with technical triplicate).

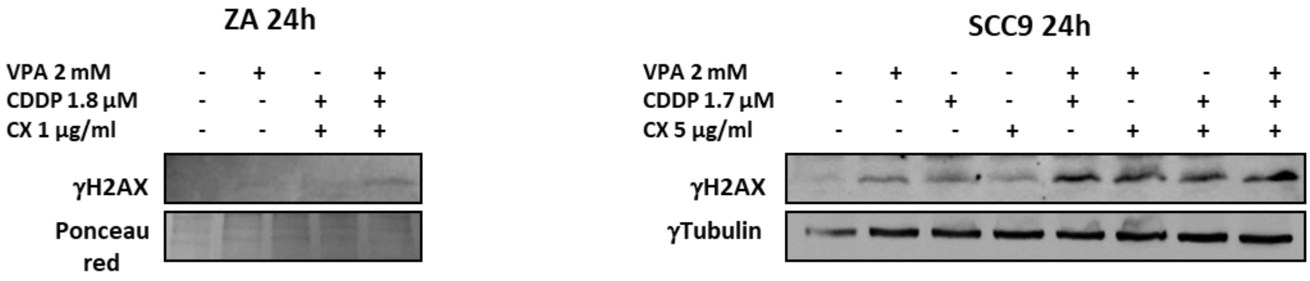

Supplementary Figure S4. VPA in combination with CDDP/CX induces early DNA damage.

Western blotting evaluation of γH2AX expression in ZA and SCC9 cells, untreated or treated as indicated for 24h. Extracts were resolved by SDS-PAGE and immunoblotted using specific antibodies. γ-tubulin or ponceau red were used as loading control.

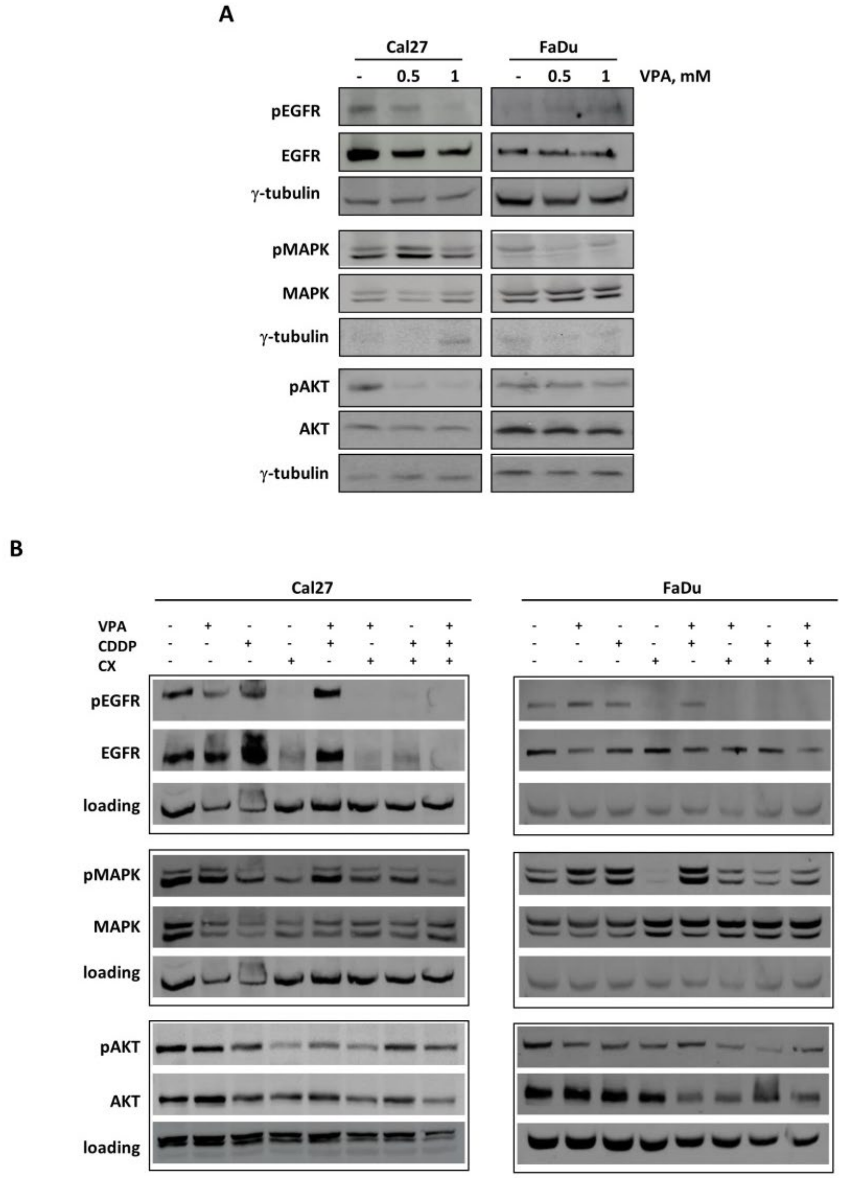

Supplementary Figure S5. Effects of VPA alone or in combination with CDDP/CX on EGFR pathway.

A. Western blotting analysis of EGFR, MAPK and AKT expression and activation in Cal27 and FaDu cells, untreated or treated at the indicated increased doses of VPA for 24h. Extracts were resolved by SDS-PAGE and immunoblotted using specific antibodies, γ-tubulin or GAPDH were used as protein loading. **B.** Western blotting analysis of EGFR, MAPK and AKT expression and activation in Cal27 and FaDu cells, untreated or treated for 24h with VPA, CCDP and /or CX at the respective IC_{50}^{96h} doses. Extracts were resolved by SDS-PAGE and immunoblotted using specific antibodies, γ-tubulin or GAPDH were used as protein loading.

4. Supplementary videos

Movie control.avi

Supplementary videos S1. Movie control

Cal27-GFP/Luc not treated spheroids were acquired on Zeiss Z1 LSFM and representative spheroid volume 3D reconstruction were performed by Fiji software with 3D viewer plug in.

Movie VPA.avi

Supplementary videos S2. Movie VPA

Cal27-GFP/Luc VPA treated spheroids were acquired on Zeiss Z1 LSFM and representative spheroid volume 3D reconstruction were performed by Fiji software with 3D viewer plug in.

Movie CDDP_CX.avi

Supplementary videos S3. Movie CDDP_CX

Cal27-GFP/Luc CDDP/CX treated spheroids were acquired on Zeiss Z1 LSFM and representative spheroid volume 3D reconstruction were performed by Fiji software with 3D viewer plug in.

Movie VPA_CDDP_CX.avi

Supplementary videos S4. Movie VPA_CDDP_CX

Cal27-GFP/Luc VPA/CDDP/CX treated spheroids were acquired on Zeiss Z1 LSFM and representative spheroid volume 3D reconstruction were performed by Fiji software with 3D viewer plug in.
